# Supplementary material for: Large harvested energy with non-linear pyroelectric modules
Source: Nature. 2022 Sep 12;609(7928):718–21. doi: 10.1038/s41586-022-05069-2 (PMC9492539; doi:10.1038/s41586-022-05069-2)
Supplement: Supplementary file 1 — Supplementary notes, tables and references. [file 41586_2022_5069_MOESM1_ESM.pdf]

nature portfolio

https://doi.org/10.1038/s41586-022-05069-2

Supplementary information

Large harvested energy with non-linear pyroelectric modules

In the format provided by the authors and unedited

Supplementary Materials for

Large harvested energy with non-linear pyroelectric modules

Authors: Pierre Lheritier[1], Alvar Torelló[1,2*], Tomoyasu Usui[3], Youri Nouchokgwe[1,2], Ashwath Aravindhan[1,2], Junning Li[1], Uros Prah[1], Veronika Kovacova[1], Olivier Bouton[1], Sakyo Hirose[3], Emmanuel Defay[1*]

Affiliations:

[1] Materials Research and Technology Department, Luxembourg Institute of Science and Technology (LIST), 41 Rue du Brill, Belvaux L-4422, Luxembourg.

[2] University of Luxembourg, 2 Avenue de l'Université, Esch-sur-Alzette L-4365, Luxembourg.

[3] Murata Manufacturing Co., Nagaokakyo, Kyoto 617-8555, Japan.

These authors contributed equally: P. Lheritier and A. Torello.

*Corresponding authors. Email: alvar.torello@list.lu; emmanuel.defay@list.lu

Content

Note 1. Multilayer capacitor inner structure

Note 2. X-Ray diffraction of PST MLC

Note 3. Calorimetric measurements of PST MLC

Note 4. Reproducibility of Olsen cycles

Note 5. Olsen cycle giving 4.43 J cm^{-3}

Note 6. Leakage current in PST-MLCs

Note 7. Stirling cycles

Note 8. HARV2 experimental set-up and results

Note 9. HARV3 Olsen cycles modelling

Note 10. Heat exchange numerical modelling description

Note 11. Autonomous energy harvester

Note 12. Efficiency of Olsen cycles in PST-MLCs

Note 13. Capacitance versus temperature

Supplementary Table 1

Supplementary Table 2

Supplementary Table 3

Supplementary Table 4

Supplementary Table 5

References

Note 1. Multilayer capacitor inner structure

Here we show the characteristics of the 0.5 mm thick PST multilayer capacitor (MLC). More details are available in Nouchokgwe et al. [1] Characteristics of 1 mm thick PST-MLCs can be found in [2].

a

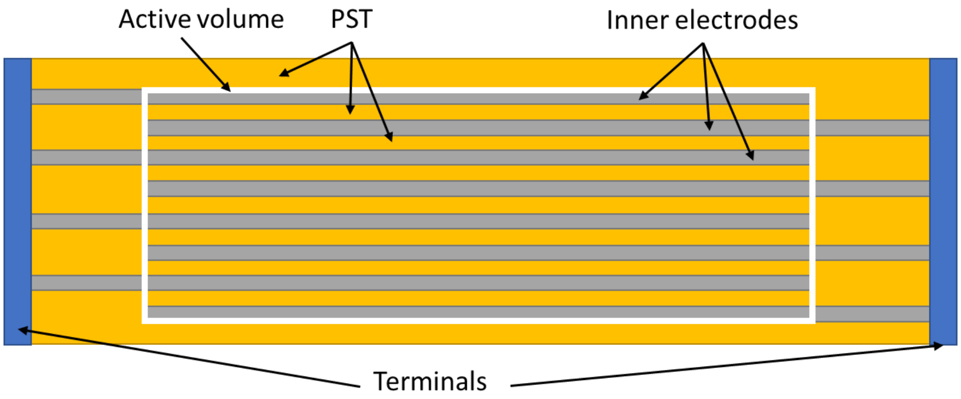

b

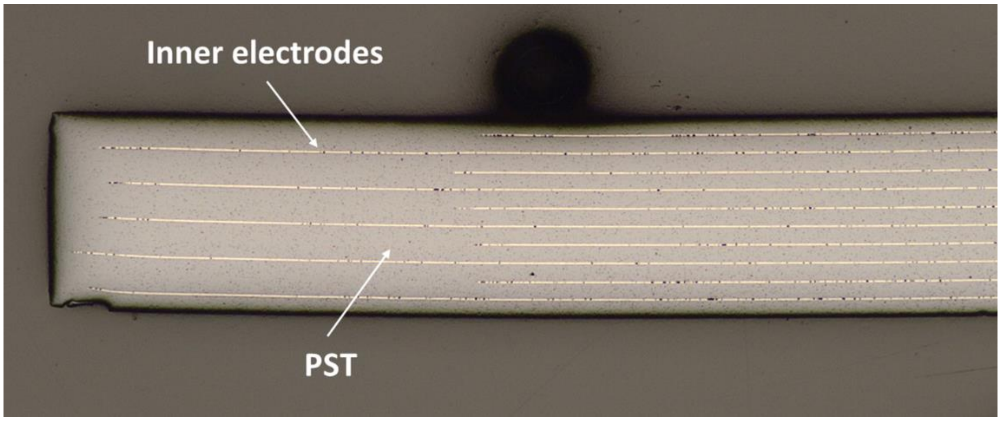

Supplementary Figure 1.1 I MLC inner structure. a) Multilayer capacitor cross section scheme showing PST, the inner electrodes made of Pt and the terminals, contacting the alternatively the inner electrodes. The white frame shows the active volume of the MLC. **b)** cross section of one multilayer capacitor observed with an optical microscope. The bright lines correspond to Pt inner electrodes, where the grey areas stand for PST

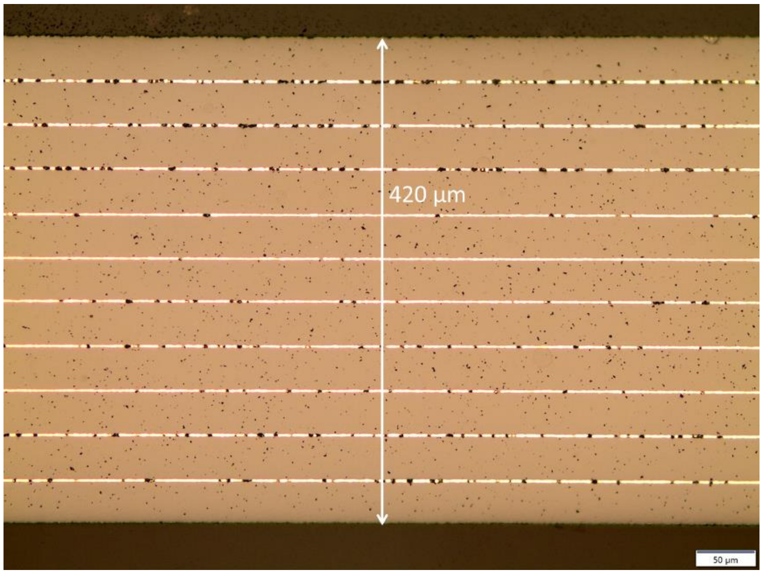

Supplementary Figure 1.2 I SEM cross section of one PST multilayer capacitor. Its average thickness is 420 µm, though we called it “0.5 mm-MLC” in the main paper for sake of simplicity.

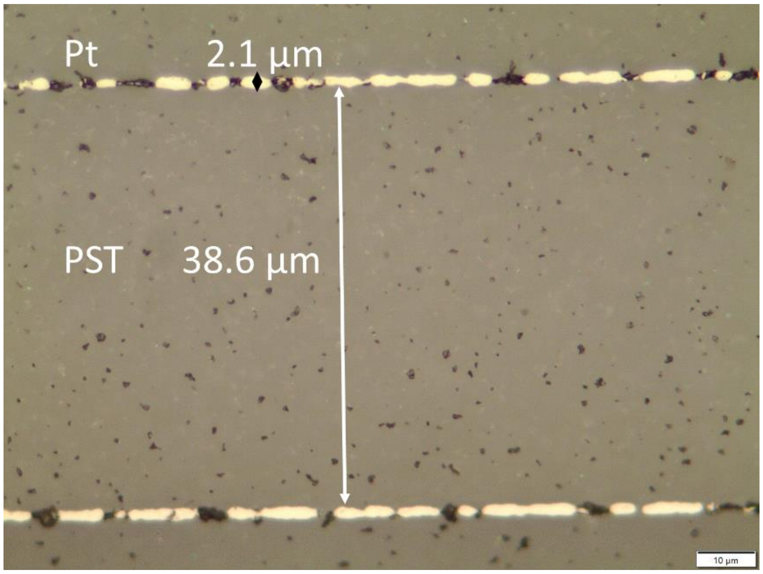

Supplementary Figure 1.3 I SEM cross section of one PST active layer and two Pt inner electrodes.

Note 2. X-Ray Diffraction of PST MLC

The B-site cation order Ω plays an important role in PST electrocaloric properties. Its extraction is obtained by comparing the (111) and (200) X-Ray diffraction peaks collected in reflexion geometry. In our experiment, the 2θ range was 15°-60°. The step increment was 0.01° with a collection duration of 2s. To be relevant, the extraction of Ω requires that PST is randomly oriented, which was obtained by crushing one MLC in order to obtain a powder. Fig. S2 shows the peaks (111) and (200) of PST powder. After [3,4], Ω is obtained by the following equation

$$\Omega^2 = \left.\frac{I_{111}}{I_{200}}\right)_{exp} \cdot \left.\frac{I_{200}}{I_{111}}\right)_{\Omega=1}$$

with $\left.\frac{I_{200}}{I_{111}}\right)_{\Omega=1}$ = 0.714. Hence, we obtained Ω = 0.75

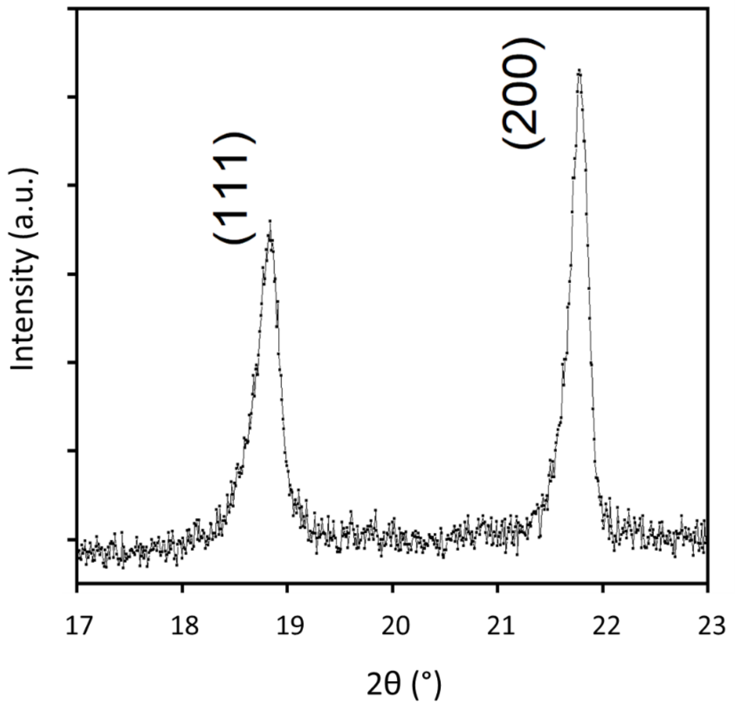

Supplementary Figure 2 I X-Ray diffraction of PST 0.5 mm-MLC powder focused on peaks (111) and (100)

Note 3. Calorimetric measurements of PST MLC

The specific heat C_p of one 0.5-mm thick MLC has been obtained with a Mettler Toledo 3 Differential Scanning Calorimeter. One MLC is too heavy to allow reliable C_p values. Therefore an 18 mg-piece of MLC has been collected to enable quantifiable measurements. We deployed the standard differential method using sapphire as a reference. The results are displayed in Fig. 3.1. We can clearly see the first-order phase transition occurring in PST at 20°C while heating. There is a thermal hysteresis because the phase transition happens at 15°C while cooling. The associated latent heat is around 550 J kg^{-1}. The background value of C_p is 300 J kg^{-1} K^{-1}.

More details are available in Nouchokgwe et al. [1]

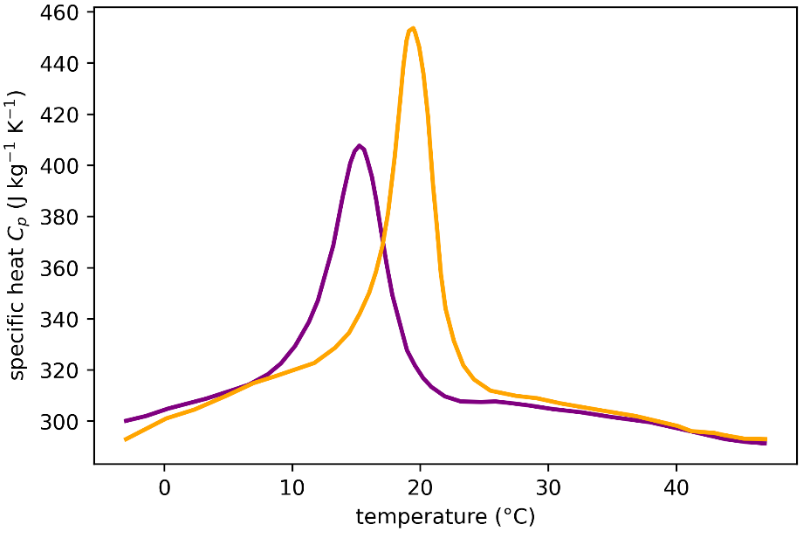

Supplementary Figure 3.1 I Specific heat C_p of a 0.5mm thick PST-MLC obtained from Differential Scanning Calorimetry. Data collected while heating (orange) and cooling (purple).

Isofield measurements

Isofield measurements are heat flow measurements at constant applied field when sweeping in temperature. These measurements presented in Fig. S3.3a, were done at a heating rate of 5 K min^{-1} on a customized DSC from Mettler Toledo. To be able to apply an electric field, the entire PST-MLC (scheme in Fig. S1.1a) of approximately 300 mg was used for this investigation. The sample mass surpasses the maximum mass (~30 mg) required to observe a good and relevant signal in DSC. Therefore, the signal measured (Fig. S3.3a) is quantitatively incorrect. However,

the position of the peak is correct as we went sufficiently slow (5 K min^{-1}) to get accurate values of the transition temperature. In a temperature range between 283 K and 323 K, constant electric fields were applied using a source meter (Keithley 2410). From isofield measurements, we constructed the ST diagram (Fig. S3.3b) on heating of PST-MLC.

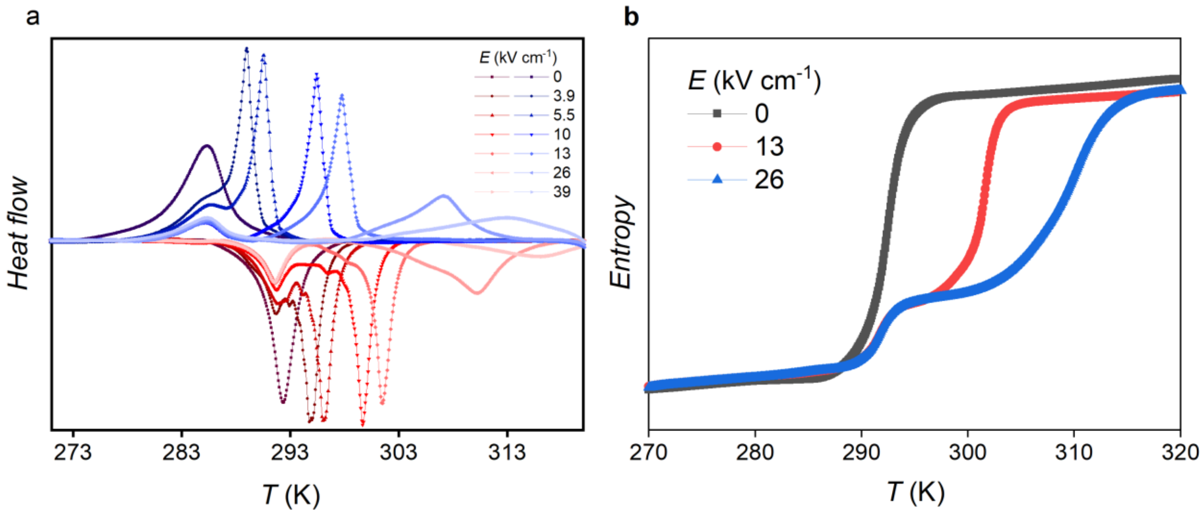

Supplementary Figure 3.2 I Isofield measurements in a PST-MLC of 300 mg. The red and blue curves are measured respectively on heating and cooling. Due to the mass of the sample which is an order of magnitude larger than the maximum required mass, the DSC signal is incorrect. Hence, no values are displayed on the y-axis in these plots. **a** Isofield measurements at different electric fields. At 0 kV cm^{-1}, we observe single peaks on heating and cooling as already seen in Fig. S3.1 showing heat flow measurements on an 18 mg piece of PST-MLC. Despite the difference in mass in both plots, one can see that the heat flow peaks at the same position at 0 kV cm^{-1}. Therefore, proving 5 K min^{-1} as a good heating rate to get accurate values of temperature in 300 mg of PST-MLC. The application of an electric field decouples the peak at 0 kV cm^{-1} into two peaks. As shown in Fig. S3.3a, one of these peaks shifts toward high temperatures with the increasing electric field. This peak (active) corresponds to the active area of the MLC where the electrodes overlap, therefore where the field is being applied (structure in Fig. S1.1a). The other peak (inactive) under applied electric field remains at the same position as the peak at 0 kV cm^{-1}. This represents the inactive region of the MLC which is unresponsive to the electric field. Moreover, the active peak sensitive to the electric field gets broader (second-order like) at higher electric fields. **b** S(T) diagram of PST-MLC on heating at different electric fields 0, 13, 26 kV cm^{-1}. This diagram was constructed from data in Fig. S3.3a using the equations described above. Under applied electric field we observed two jumps at two different temperatures. The low-temperature jump is insensitive to the electric field (inactive region of MLC) as it occurs at the same temperature as at 0 kV cm^{-1}. The high-temperature jump corresponds to the active region. This jump is shifted towards high temperatures with the increasing electric field.

Note 4. Reproducibility of Olsen cycles

Here we show reproducibility of Olsen cycles by running several cycles under the same conditions consecutively.

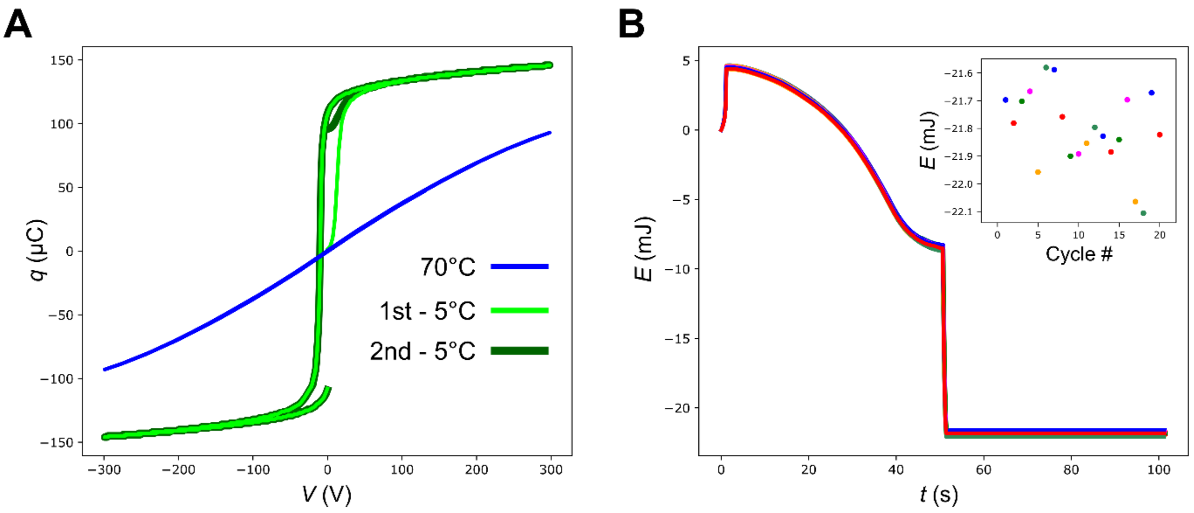

Supplementary Fig. 4a Charge *q* of one 1 mm PST-MLC as a function of the applied voltage *V* at 70°C (blue), first cycle at 5°C when the sample is not poled (light green) and second cycle at 5°C (dark green). **b** 20 cycles energy curve of 1 mm PST-MLC as a function of time with isofield legs at 0 and 80 kV cm^{-1} (300 V) and isothermal legs at 5°C and 70°C (time resets after each cycle). The inset shows the energy harvested at the end of each cycle.

Note 5. Olsen cycle giving 4.43 J cm^{-3}

Here we show an Olsen cycle in a 0.5 mm thick PST-MLC showing the highest energy density harvested (4.43 J cm^{-3}). The total energy harvested is 74.9 mJ (0.0749 J). The total active volume of PST is 16.9 mm^3 (0.0169 cm^3).

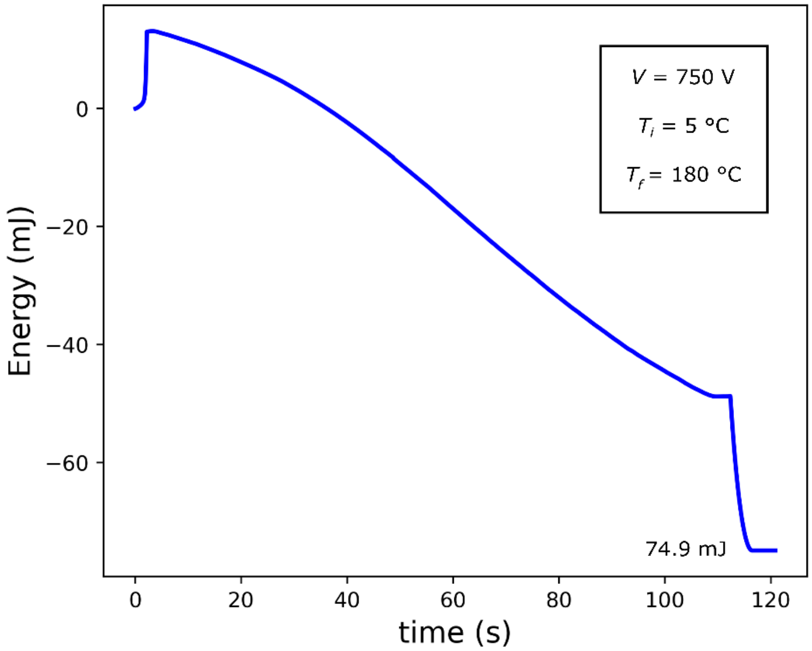

Supplementary Figure 5. Olsen cycle in a 0.5 mm thick PST-MLC. The applied voltage is 750 V and the isothermal legs 5 °C and 180 °C. The total energy harvested is 74.9 mJ, which corresponds to an energy density of 4.4 mJ cm^{-3}.

Note 6. Leakage current in PST-MLCs

We measured the leakage current of a 0.5 mm thick PST-MLC at constant field (195 kV cm^{-1}, 750 V) and different temperatures (Fig S6.1). The raw experimental data was fitted with Curie-von Schweidler empirical law (Fig. S6.2) and finally the leakage current plotted as a function of temperature (Fig. S6.3).

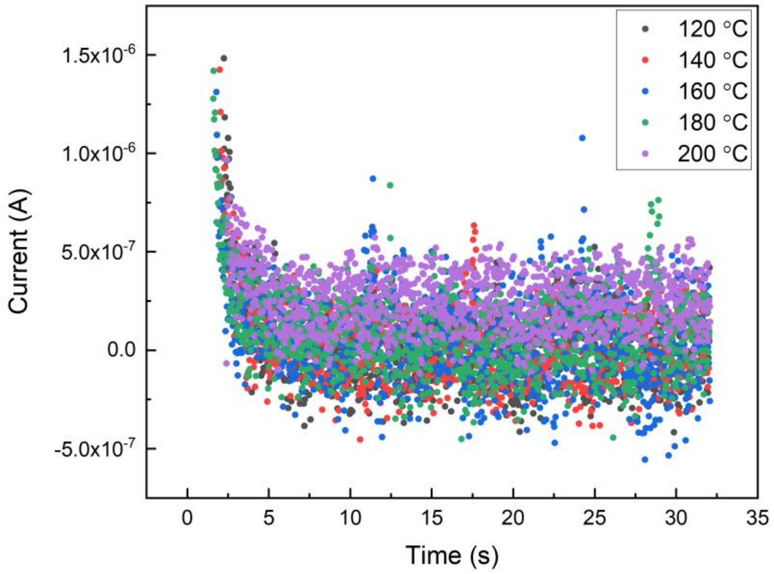

Supplementary Figure 6.1. Leakage current of a 0.5 mm thick PST-MLC at the applied voltage of 750 V and different temperatures (120 to 200 °C).

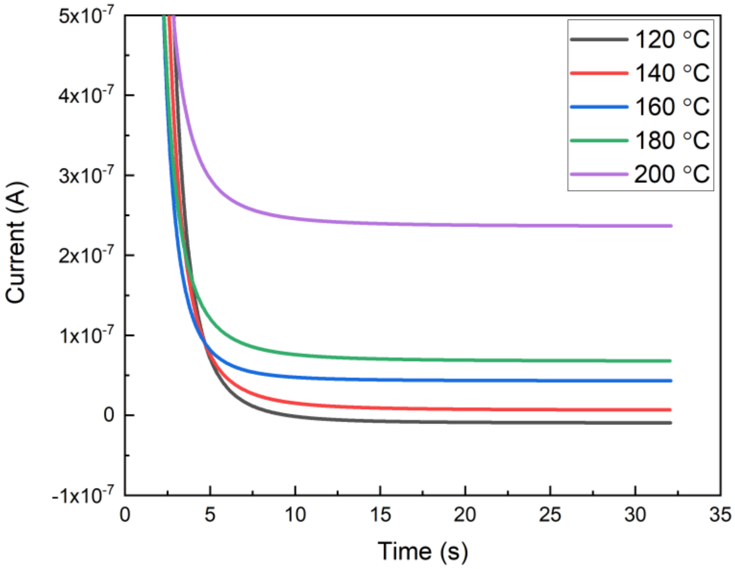

Supplementary Figure 6.2. Leakage current time evolution after fitting the experimental data with Curie-von Schweidler empirical law [5].

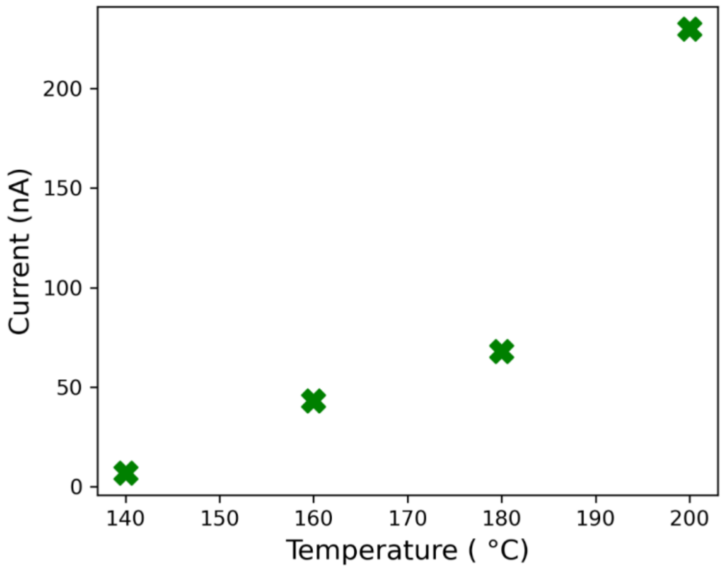

Supplementary Figure 6.3. Leakage current in 0.5 mm PST-MLCs as a function of temperature for an applied electric field of 750 V.

Theses graphs show that the leakage current of our PST-MLC sample is <10^{-7} A at 750 V applied and temperatures lower than 180 °C. Note that for temperatures T<140 °C, the precision of our sourcemeter is not enough to capture the leakage current.

Note 7. Stirling cycles

Here we show actual Stirling cycles: in 1 mm thick PST-MLCS (Fig. S7.1), mimicking the conditions of the *DE*-loops from Figure 1b; and in 0.5 mm thick PST-MLCs (Fig. S7.2), with different applied initial voltages and temperature spans.

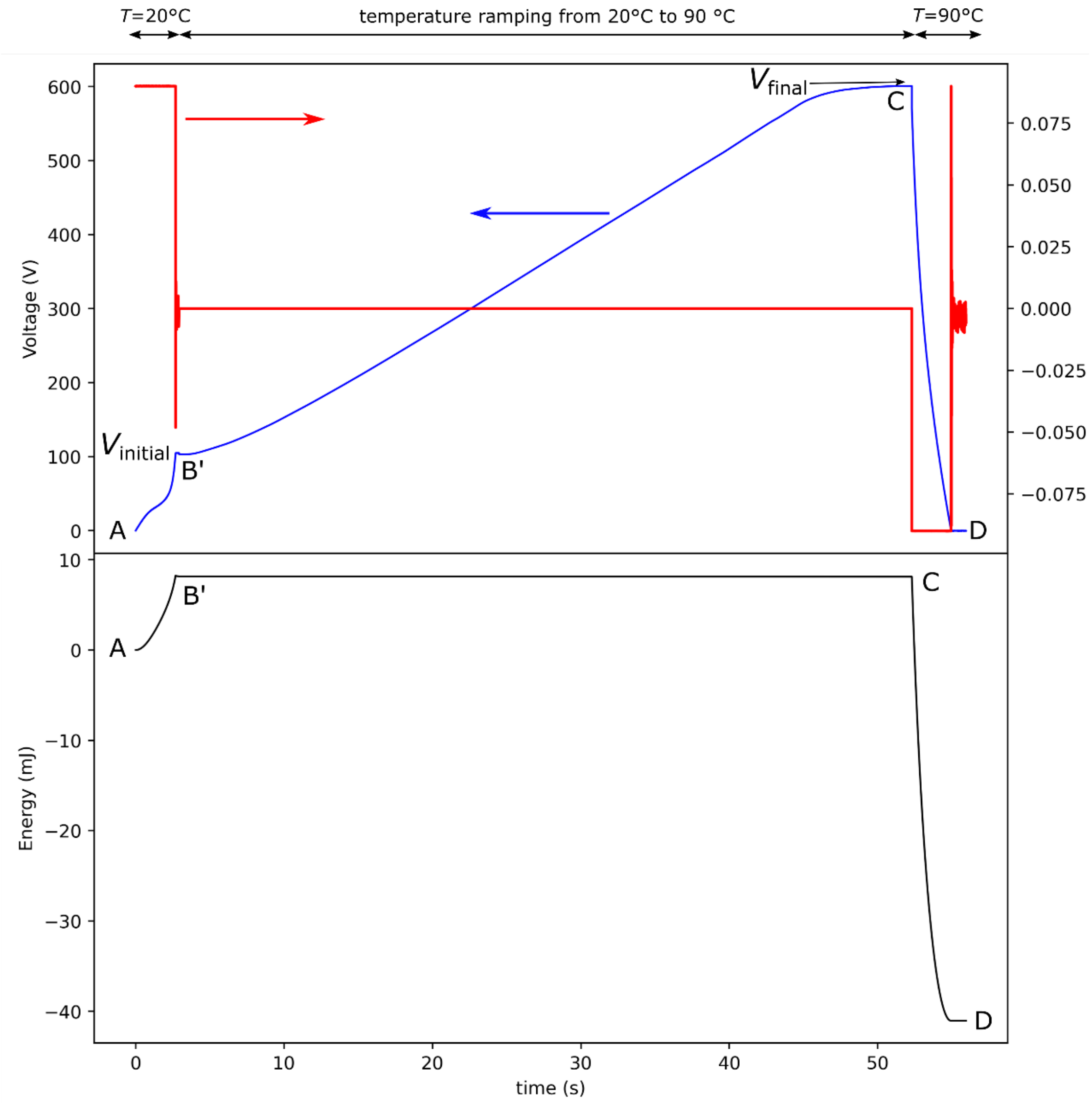

Supplementary Fig. 7.1. Real Stirling cycle for a 1 mm thick PST-MLC. Starting temperature is T_i = 20°C, and input voltage V_i = 105 V and compliance current *I* = 90 μA (AB'). The energetic cost of this step is 8.13 mJ. At B', the circuit is open (*I* = 0 A) and the temperature is ramped to 90 °C. As a result of the pyroelectric effect, the voltage in the sample increases to 600 V at C. Because the current is 0, the energy in the sample remains the same. At C, the sample is discharged (*V* = 0) at the same compliance current (*I* = 90 μA), giving back 49.16 mJ (D). Subtracting the initial 8.13 mJ required to charge the PST-MLC, 41.03 mJ of energy have been

harvested. To conclude the cycle, the sample is cooled down to its initial temperature (20°C) in the leg DA.

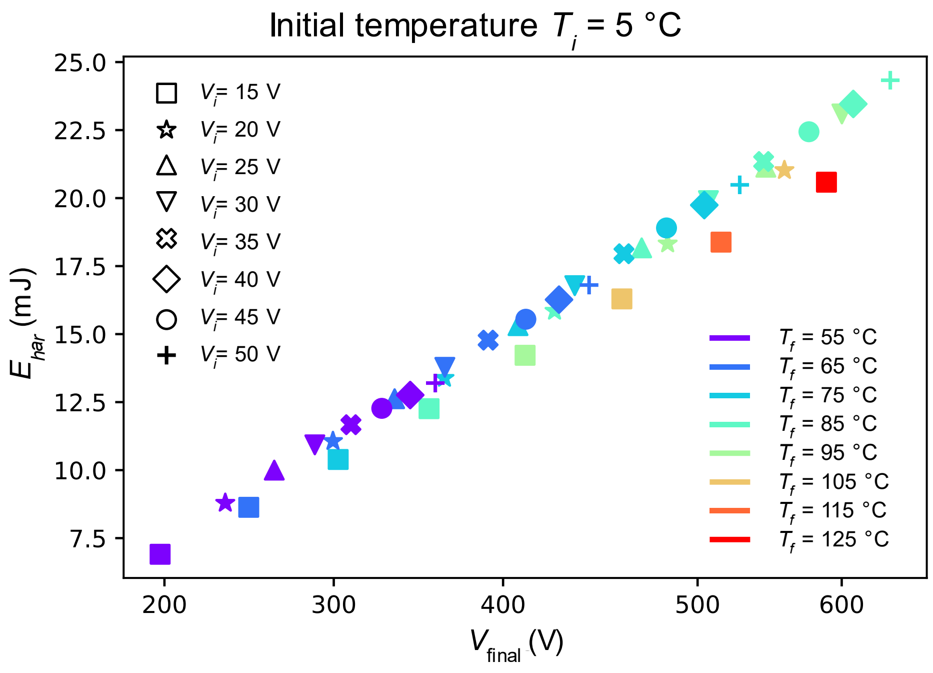

Supplementary Fig. 7.2. Energy harvested with Stirling cycles for a 0.5 mm thick PST-MLC as a function of the final voltage. The initial temperature of the cycle is 5 °C for all points, and the initial voltage takes values from 15 to 50 V, as the symbols in the legend show. The color legend indicates the final temperature of each cycle/point.

Note 8. HARV2 experimental set-up and results

The experimental set-up and design of HARV2 is shown in Fig. S8.1.

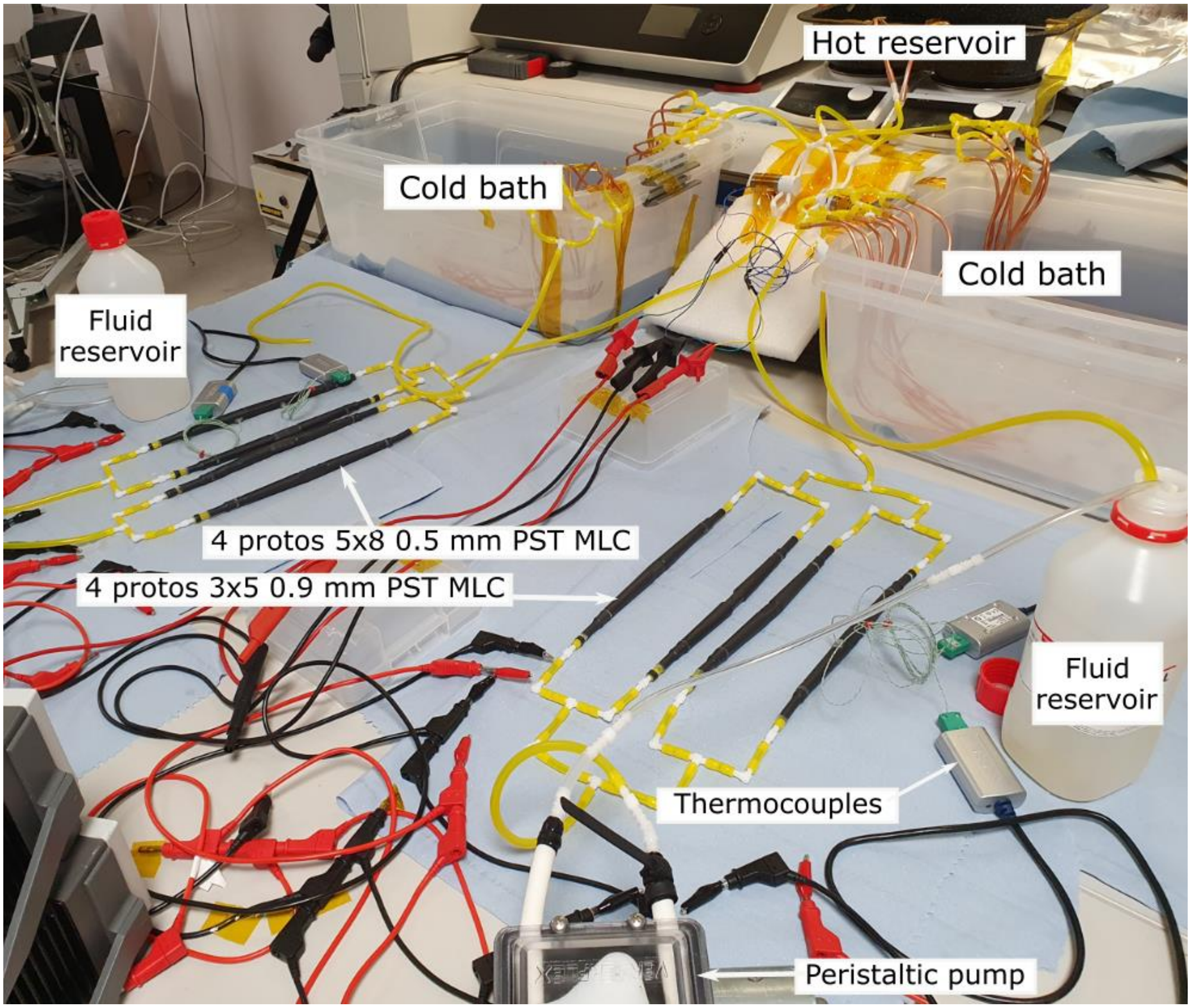

Supplementary Figure 8.1 Experimental set-up and design of HARV2. It consists of 8 prototypes made of PST-MLCs, 4 of them (left-hand side) are structured in a parallel-plate matrix of 8 rows x 5 columns of 0.5 mm thick PST-MLCs (HARV2_160 subunit), and the other 4 (right-hand side) are in a parallel-plate matrix of 5 rows x 3 columns of 1 mm thick PST-MLCs (HARV2_60 subunit). The fluid circuit is divided into two circuits (yellow tube and white plug-in connectors), each with their own cold bath and fluid reservoir. The hot reservoir is shared by the two fluid circuits, with no fluid returning so that its temperature it is not altered. Two pinched valves select accordingly whether to pump from the cold bath (to cool down the PST-MLCs prototypes) or the hot reservoir (to heat them up). The 8 prototypes are connected electrically in parallel and are charged and discharged from a sourcemeter. Thermocouples are added at inlet and outlet of 2 prototypes to monitor temperature. Sourcemeter, valves, thermocouples and pump are governed and

synchronized with a python script, so the system operates Olsen cycles. All 8 prototypes were operated simultaneously.

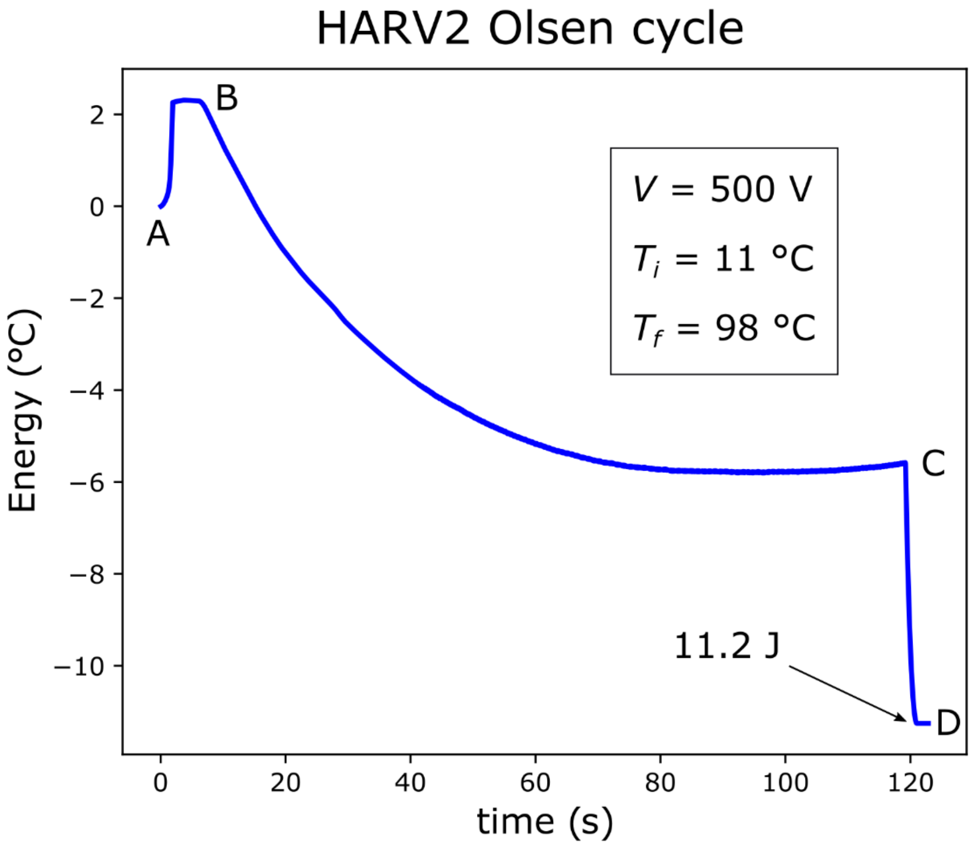

Supplementary Fig. 8.2 Energy of the Olsen cycle run in HARV2 vs time. In cycle's step A, the PST-MLCs in HARV2 are charged to 500 V (E = 130 kV cm^{-1}) at a current of 15 mA and temperature T_i = 11 °C. The energetic cost for that is 2.3 J. At B, the hot fluid starts circulating and heats up the PST-MLCs. As a result, some HARV2 starts harvesting some energy, reaching -6 J at C, when the PST-MLCs reach thermal equilibrium with the hot fluid at a temperature of 98°C. Right after, PST-MLCs are discharged, and the energy harvested value reaches 11.2 J (D). At this point, the cold fluid starts circulating, cooling down the temperature of the PST-MLCs so that another cycle can be started again.

Note 9. Olsen cycles in HARV3

Olsen cycle in one single 0.5 mm-thick PST-MLC inserted in one prototype, similarly to what is described for HARV1 and HARV 2. This specific small prototype has been fabricated to estimate how fast the heat exchange can happen with these MLCs and the dielectric fluid we have used all along this study.

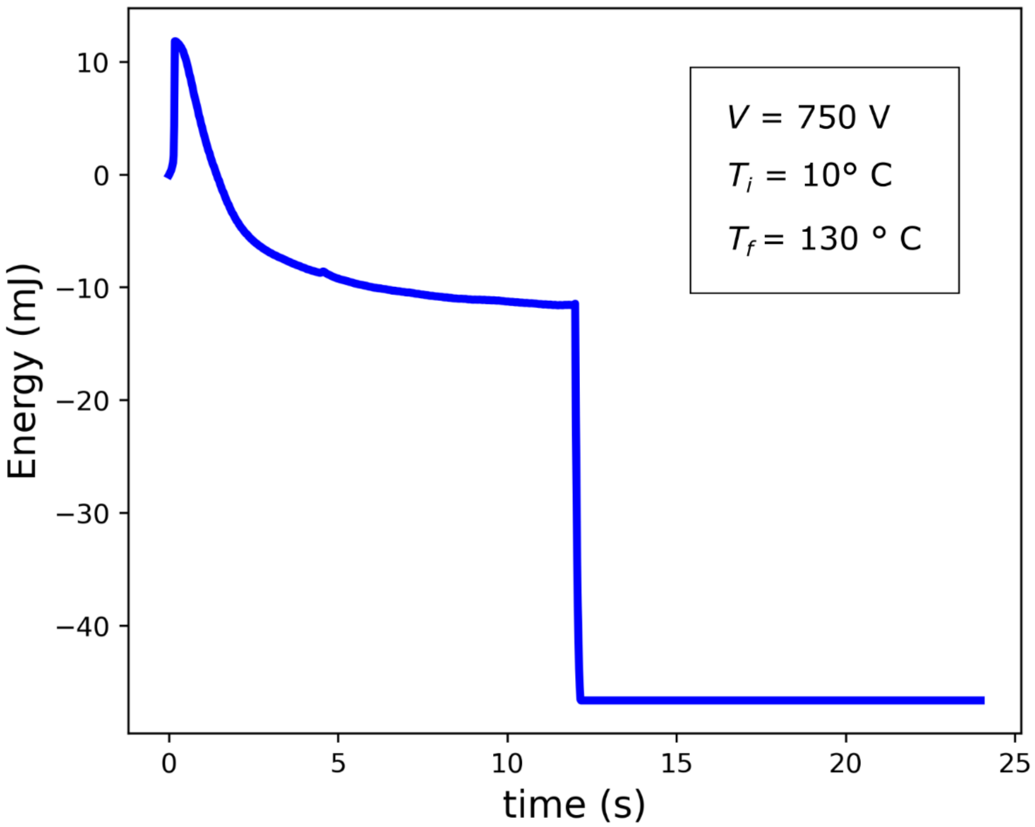

Supplementary Figure 9. Energy time evolution of HARV3 running under Olsen cycles. The applied voltage is V = 750 V (E = 195 kV cm^{-1}) and the temperature span ~120 °C (10°C to 130°C). The harvested energy is 47 mJ and the period cycle 24 s. This indicates a cycling power of 1.95 mW (per each 0.5 mm thick PST-MLC).

Note 10. Heat exchange numerical modelling description

Exploration of better power results was carried out with finite elements method COMSOL Multiphysics software, where heat transfer and fluid dynamic modules were coupled in the non-isothermal laminar flow module. Simulations consisted of a time dependent 2D representation of the macroscopic harvester prototype in Fig. 2a (main text). The geometry simulated included only the fluid column and the 28 1 mm thick PST-MLC matrix (Fig. S10.a). In the simulations, the fluid from the hot reservoir (set at 115 °C) is sent through the PST-MLC matrix, set initially at 10 °C, at the desired flow rate. To help the numerical integration, a 2 second linear ramp of the velocity is set first. At this t = 2 then, the fluid inlet becomes 115 °C, simulating the time it takes the fluid to travel from the reservoir to the harvester, which is approximately 2 s. We call this time as waiting time τ_w. To start with, correspondence with the experimental value (54.4 mW) was verified by computing the time that the average temperature of the PST-MLCs thermalized with the temperature of the incoming fluid (thermalization time τ_t) plus the already defined waiting time.

To match the experimental conditions, the fluid used initially in the simulation was silicone oil and the PST-MLC matrix consisted of 7 columns x 4 plates. The τ_t obtained after running the simulations was of 25.2 s. Adding the 2 s of τ_w, this gives as a cycle period ($2\tau_w + 2\tau_t$) of 54.4 s, very close to the 57 s of the experiment. Afterwards, new simulations were run for different PST-MLC thickness (Fig. S10b) and configurations. These configurations explored consisted of 1st) using water as a fluid and keeping the same matrix configuration of 7 columns x 4 plates, 2nd) using water as a fluid and reversing the PST-MLC matrix to 4 columns per 7 rows and 3rd) using water as a fluid, reversing the PST-MLC matrix to 4 columns per 7 rows and $\tau_w = 0$. As a matter of fact, the porosity of the harvester was kept constant in all the points (50%), meaning that the fluid slit was changed accordingly to the PST-MLC thickness change. The fluid flow in the simulation was set initially equal to the experimental one (112 mL min^{-1}). In the reversed PST-MLC matrix configuration (4 columns x 7 rows), the fluid flow was increased accordingly so that the fluid velocity through the PST-MLC channels was always the same (112 x (7/4) = 196 mL min^{-1}). The properties of the materials as well as the complete geometry dimensions can be consulted in Tables S2-S4. To calculate the simulated power, 3.1 J were assumed to be harvested at each case since in all cases the applied voltage is assumed to be 750 V, the temperature span 115 K and the total PST-MLC mass remains the same. This energy value of 3.1 J was divided then by the cycle period obtained, which is $2\tau_w + 2\tau_t$.

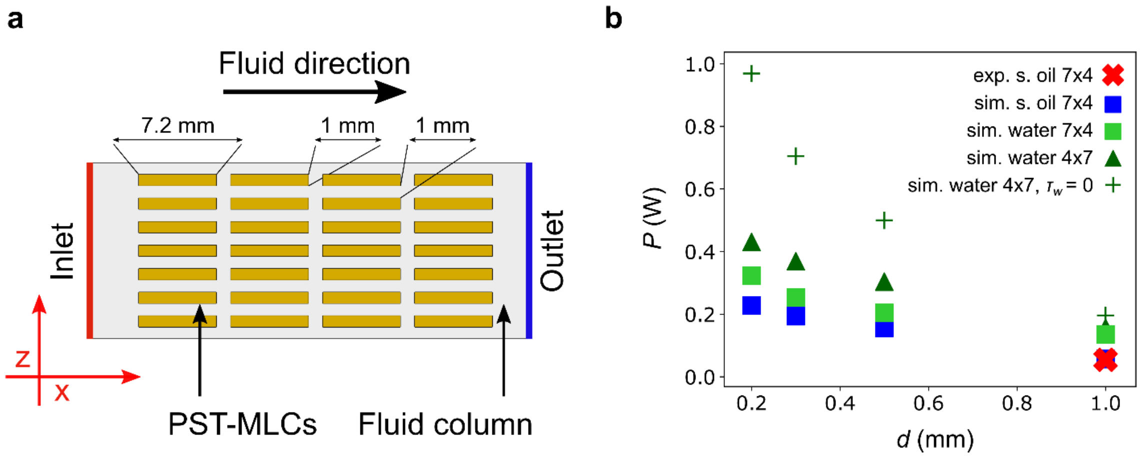

Supplementary Figure 10. a) Sketch of the geometry simulated in COMSOL Multiphysics. A fluid inlet is placed at the red wall and an outlet at the blue wall, providing the fluid motion. The fluid column is made of the chosen fluid, either silicone oil or water (see table S3 for material properties). The configuration in the figure consists of a PST-MLC matrix of 4 columns and 7 rows, which correspond to the green triangles in panel **b**. More details about the structure and dimensions of the experiment and simulations are given in tables S2 and S4. **b)** Power of the harvester prototype as a function of the PST-MLC thickness. The red cross represents the experimental point in Fig. 2b where 3.1 J were harvested in 57 s period using silicone oil as the heat exchange fluid and a parallel plate matrix of 7 columns and 4 rows. The blue square represents the corresponding values obtained with our FEM simulations with the same fluid and PST-MLC matrix. The green squares represent the corresponding values when using water as a heat exchange fluid and the same PST-MLC matrix. In the dark green triangles, water was used as a heat exchange fluid, but the PST-MLC matrix was reversed to 4 columns and 7 rows. In the dark green pluses, water was used as a heat exchange fluid, the PST-MLC matrix consisted of 4 columns and 7 rows and the time the fluid needs to travel from the reservoir to the harvester τ_w was neglected ($\tau_w = 0$).

Note 11. Autonomous energy harvester

The main objective of this autonomous system is to spontaneously harvest energy by thermal cycling when the pyroelectric material reaches a pre-determined temperature. The energy harvested from the PST-MLCs is efficiently recovered and utilized for charging our system for the subsequent cycles. Figure S11.1 is the block diagram of our harvester.

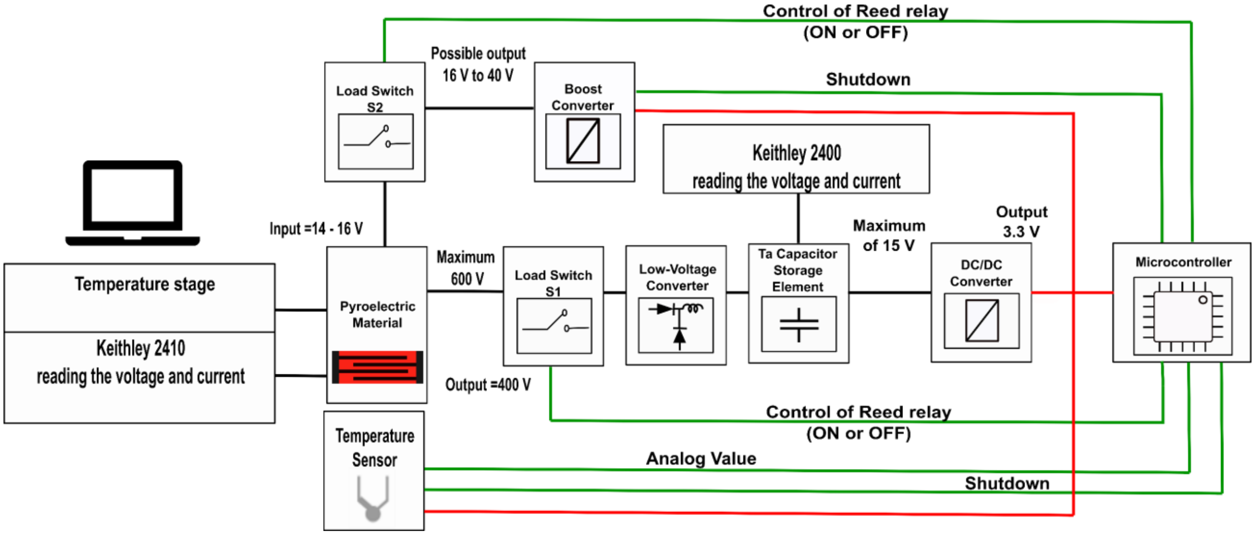

Supplementary Figure 11.1. Global diagram of the autonomous pyroelectric energy harvesting system.

Electronic components in the autonomous system

Two PST-MLCs (10.4 x 7.2 x 0.5 mm³ connected in parallel) act as the heat harvester that will experience a Stirling cycle. Two reed relays (S2-05EU / S2-03PU) act as load switches to control the flow of current in the circuit. To step down the voltage from the harvester that reaches hundreds of volts, we employ a low voltage converter made of two diodes (BYW56-TR), an inductor (1140-122K-RC) and a multilayer storage capacitor (FA22X7R1E226MRU06) in the system (described later in Fig. 11.3). In addition, we have a DC/DC converter (LTC 3588-1) to deliver a regulated DC output voltage at 3.3 V. The system also embeds a low power consuming microcontroller (ATtiny 45), a temperature probe (TMP36FSZ) and a boost converter (LT8410) to ensure the autonomous charging and discharging of the system as a function of temperature. There is no battery in all this system.

Description of the autonomous pyroelectric energy harvesting system

For all this description, please refer to Figure 11.1. A Keithley 2400 is used to initially charge the storage capacitor (Ta capacitor storage element in Fig.11.1) at room temperature. This initial

energy initiates the electronic components in the autonomous system. Once this initial energy is provided, the system runs autonomously.

The energy from the storage capacitor will tread through the LTC 3588-1 voltage regulator, which delivers a regulated DC voltage of 3.3 V. The output of LTC 3588-1 serves as the input for the ATtiny 45 microcontroller (MC). Note that the minimum operating voltage of the LTC is 4 V. Hence, the voltage in the storage capacitor must always be greater than 4 V for the functioning of the autonomous pyroelectric energy harvesting system.

ATtiny 45 MC consumes very little power (0.7 μA at a clock frequency of 1 MHz and 0.2 μA / 3.3 V during the low power down). The switching, boost and shutdown conditions for the MC were programmed by Arduino IDE. The MC gathers temperature data from the temperature probe every 4 seconds.

In order to mimic a proper Stirling cycle, the temperature of the pyroelectric PST-MLCs is varied using a Linkam stage (cf Fig. 11.1). If the temperature of PST is below the threshold temperature set in the MC, the MC will close the load switch S2 (reed relay S2-05EU / S2) between the boost converter and the PST-MLC. The boost converter will step up the output voltage from the LTC 3588-1 and deliver a DC voltage between 16 and 40 V to charge the PST-MLC. After the charging event, the switch S2 is opened, and a Stirling cycle is executed on the PST-MLC (temperature increase at constant charge, meaning open circuit conditions for PST). An automated system was created using Python programming to control the Linkam stage, Keithley 2400 and Keithley 2410. Hence, we can simultaneously execute the Stirling cycle and monitor the voltage in the system.

If the temperature of the material is above the set threshold temperature in the MC, the MC will spontaneously discharge PST-MLCs in the storage capacitors through the low voltage converter by closing the load switch S1 (reed relay S2-05EU / S2). Indeed, the voltage from PST-MLC (V_{final}) must be stepped down using a low voltage converter before storing the energy in the capacitor. For this system to be sustainable, the energy harvested and stored in the capacitor must be greater than the energy required for running the autonomous system. Fig. 11.2 gathers pictures of the entire autonomous sensor set-up.

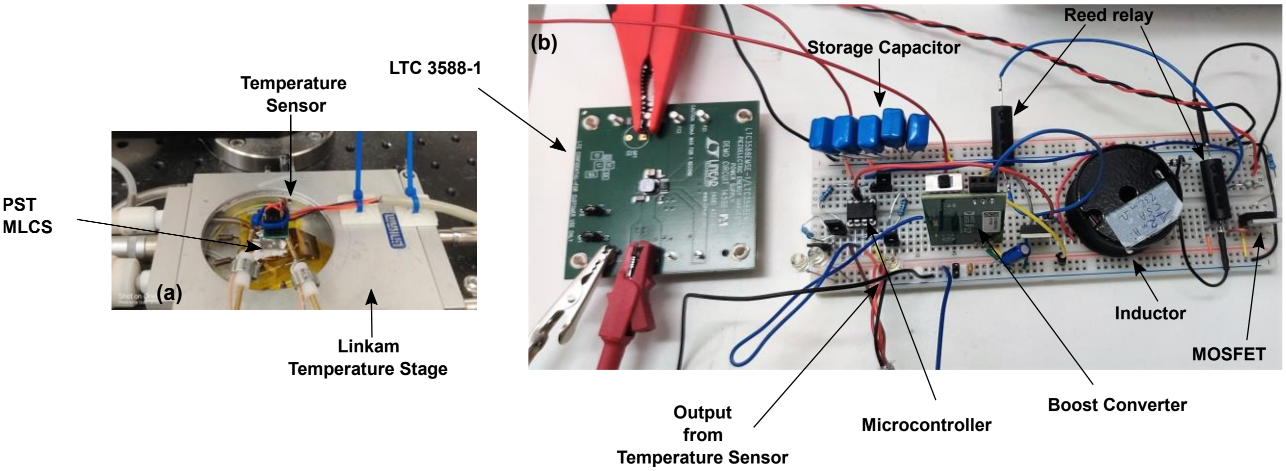

Supplementary Figure 11.2. Experimental set-up of the autonomous sensor: a) Linkam temperature stage with the PST-MLCs and temperature sensor and (b) electric circuit with the storage capacitors, reed relay, inductor, microcontroller and boost converter.

Low voltage converter

The low voltage converter steps down the voltage from the pyroelectric material and delivers a low voltage output. It consists of a reed relay, an inductor and two diodes (cf Fig. 11.3). A low voltage converter operates by storing the energy in the inductor in the form of a magnetic field. The working mechanism of the low voltage converter is akin to the buck converter, which is a DC/DC step-down converter.

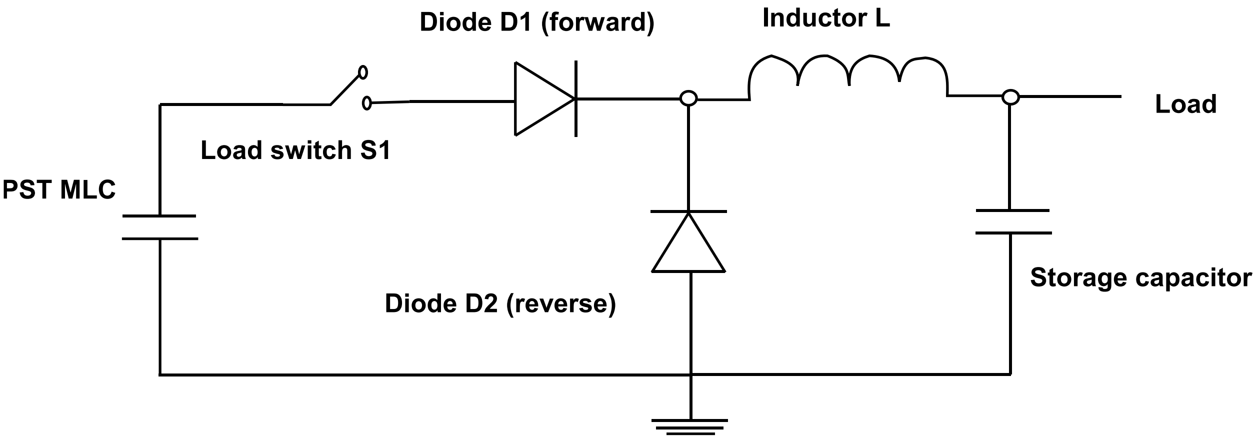

Supplementary Figure 11.3. Schematics of the low voltage converter.

When the switch S1 is closed, the current from PST-MLC passes through the diode D1 towards the inductor. The inductor opposes the changing current by creating a voltage drop across its terminals. The energy is stored in the inductor and then in the capacitor. All this time, the diode D2 is reverse biased, and thus prevents flow of current. When the switch S1 is opened, the polarity

of the inductor reverses due to the collapsing field and the diode D2 becomes forward biased. In this way, the energy from the PST-MLC is efficiently stepped down and stored in the capacitor.

The next step was to estimate the energy recovered in the storage capacitor after stepping down the voltage from PST-MLC using the low voltage converter. For this study, the Stirling cycle was executed for five different temperature spans. The input voltage and initial temperatures were 20 V and 5 °C respectively. One tantalum electrolytic capacitors (*C* = 10.5 μF and voltage rating of 16 V) was used as storage capacitor. The energy in the storage capacitor was calculated using $\frac{1}{2}CV^2$. The results of these five Stirling experiments with increasing final temperatures are displayed in Fig. 11.4. The ratio between the electrical energy present in the MLC after harvesting and the electrical energy transferred in the Ta capacitors if displayed in red as right hand-side Y-axis. With this system, we recover between 75 and 80% of the electrical energy harvested in PST-MLC. Note that the voltage across PST-MLC varies between 250 V and 550 V and that the one across the storage capacitor does not overtake 16 V. This is extremely important because the LTC cannot accept input voltage larger than 20 V. This low voltage converter therefore plays a paramount role in this autonomous energy harvester.

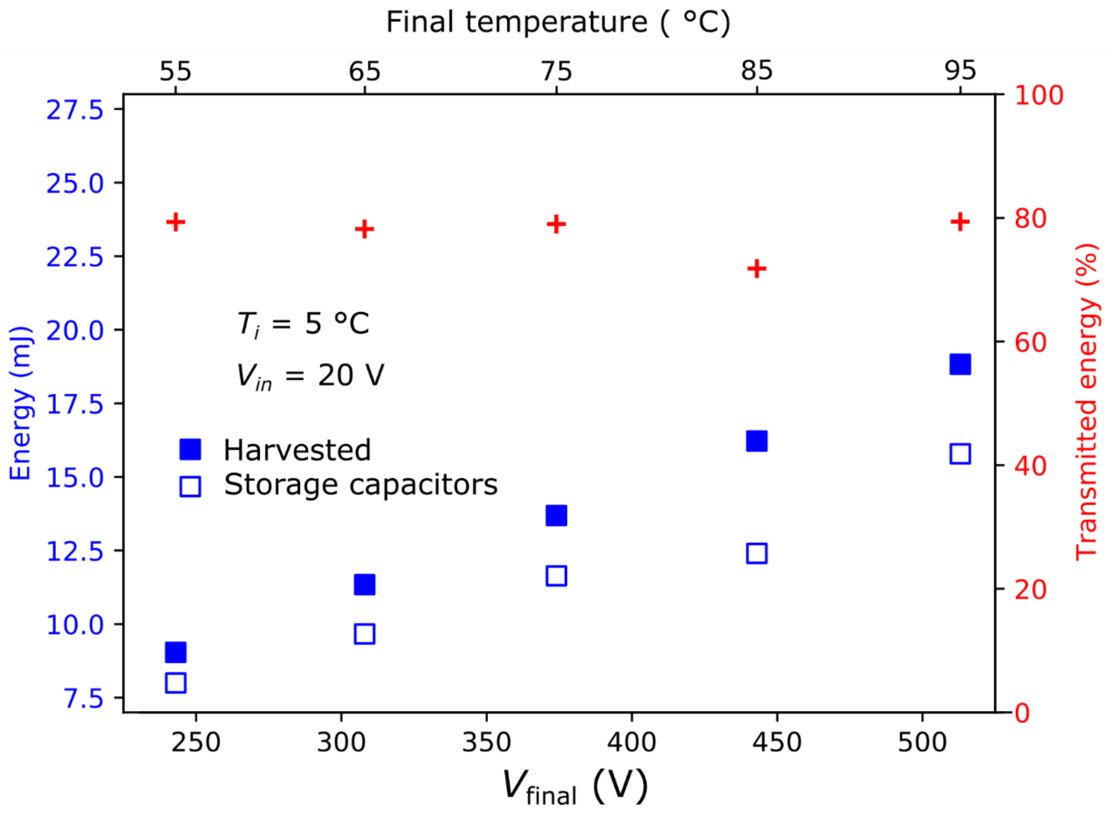

Supplementary Figure 11.4. Energy harvested from the PST-MLC (full blue squares) and energy stored in a Ta storage capacitor after transfer from the PST-MLC (empty blue squares) when Stirling cycle was executed for different temperature spans. The initial temperature is always 5 °C and the final temperatures of the Stirling cycle varies from 55°C to 95°C. V_{in} is the initial voltage applied to the PST-MLC at the beginning of the Stirling cycle (starting at low temperature). The right-hand Y-axis is the ratio between the harvested and stored energies, which corresponds to the percentage of energy collected after this low voltage conversion. The top X-axis is the final temperature of the Stirling cycle. And the bottom X-axis is the final voltage reached across the PST-MLC. Note that values as high as 550 V can be reached, while the initial voltage is only 20V.

Results of the autonomous energy harvester

A Stirling cycle of the entire autonomous energy harvester described in Fig. 11.1 was carried out for a temperature span of 90 °C, from -5 to 85° C. The threshold temperatures in the MC was programmed at 5°C and 60 °C. Only when the temperature is away from these limits, the boost and shutdown event occur spontaneously. In this study two PST-MLCs (10.4 x 7.2 x 0.5 mm^3) connected in parallel have been used as the thermal energy harvester. Firstly, the storage capacitor (C = 500 μF) was charged to 9 V at 15 °C to initiate the first cycle. Then the temperature of the Linkam stage is decreased to -5 °C for a dwell time of 8 s. Then, the MC closes S2 between the boost converter and harvester after having detected the temperature below 5°C. The boost converter collects energy from the storage capacitor via the LTC 3588-1 (at 3.3 V then) and delivers a DC voltage of 15 V to charge the PST-MLCs (initial voltage of the Stirling cycle). Following the charge event, the material is open-circuited and the temperature of the Linkam stage is raised to 85 °C for a dwell time of 13 s. Thanks to the pyroelectric effect of PST, the voltage of the PST-MLCs reaches 400 V at 85 °C, as visible in Fig. 11.5, right hand side panel.

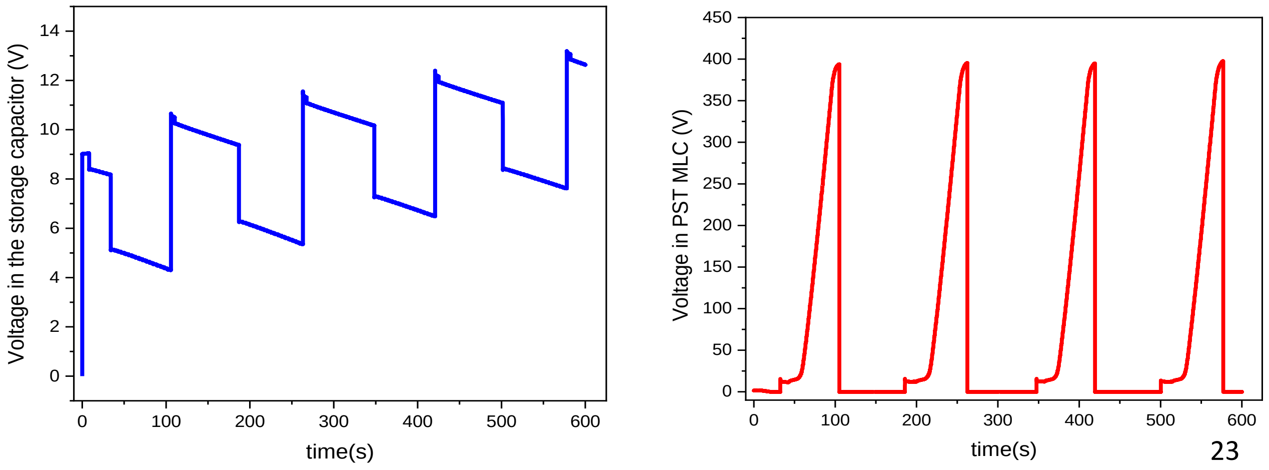

Supplementary Figure 11.5. Voltage time evolution in the storage capacitor (left) and the voltage in the two 0.5 mm-thick PST-MLCs (right).

Then, the MC automatically discharges the sample at 85 °C (second threshold reached) and the voltage from PST-MLCs is stepped down through the voltage converter and the corresponding energy stored in the storage capacitors. The voltage of the storage capacitors thus increases from 4.3 V to 10.8 V, as shown in Fig. S11.5, left hand side panel. Note that one Stirling cycle is executed in 160 s. At the end of four Stirling cycles, the voltage of the storage capacitor reaches 13.2 V (corresponding to 42 mJ). Hence, an energy gain of 22 mJ was observed after executing four Stirling cycles. Figure S11.5 unambiguously shows that the voltage of the storage capacitors keeps on increasing over the succession of the four Stirling cycles. This proves that our system is sustainable in these conditions.

We also performed the same experiment with two (10.4 x 7.2 x 1 mm^3) thick PST-MLCs. Similarly, several successive Stirling cycles were executed from -5 to 85° C, as shown in Fig. S11.6. All the other parameters were the same except the initial voltage that was 14 V at the beginning. Here, we executed 16 Stirling cycles and we observed the same increasing trend in the voltage of the storage capacitor over the cycles.

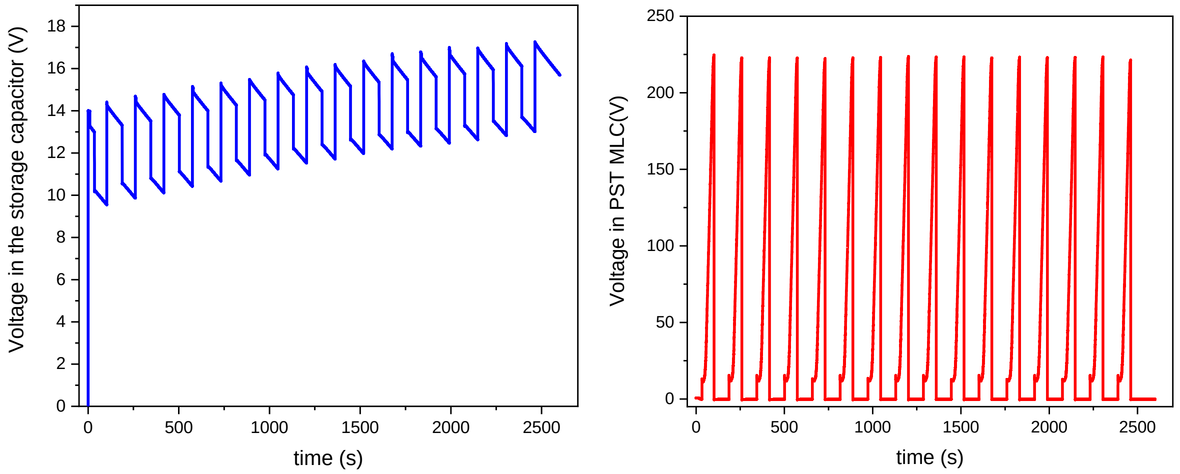

Supplementary Figure 11.6. Voltage time evolution in the storage capacitor (left) and in the two 1mm-thick PST-MLCs (right).

Note 12. Efficiency of Olsen cycles in PST-MLCs

The figure of merit η is defined as the ratio of harvested electrical energy density N_d over input heat density Q_{in}

$$\eta = \frac{N_d}{Q_{in}} \quad (2)$$

where N_d is calculated from integrating the area between two *DE*-loops at the two oscillating temperatures of a given Olsen cycle (Fig. S12.1). These *DE*-loops have been measured experimentally with a Keithley sourcemeter 2410. The electric displacement field *D* is calculated by numerical integration of the current supplied over time (electric charge) and dividing it by the active area (0.489 cm^2) and number of active layers (9) of a 0.5 mm thick PST-MLC (Table S5).

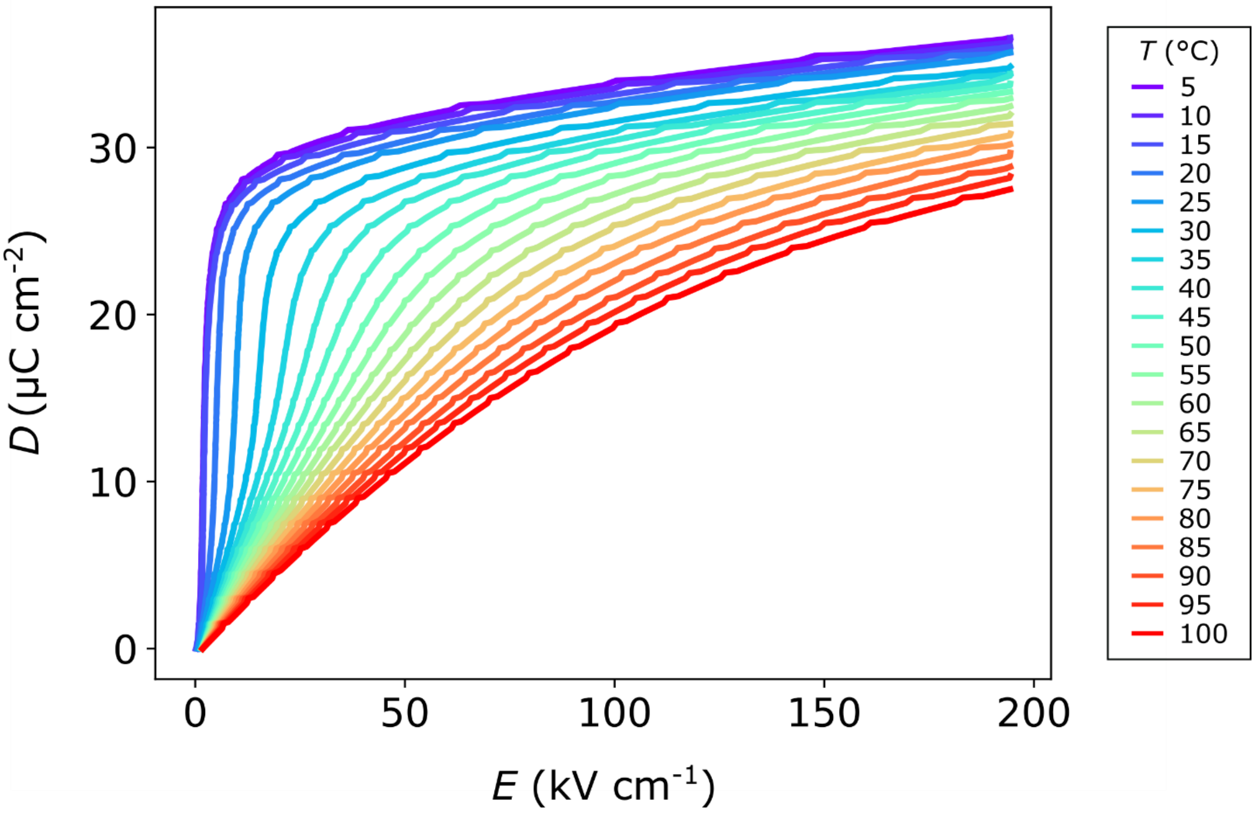

Supplementary Figure 12.1 Electric displacement field *D* – electric field *E* loops for a 0.5 mm thick PST-MLC at temperatures ranging from 5 to 100 °C.

The amount of heat absorbed by the material has two contributions: 1) the Olsen heating leg BC, in which the sample undergoes a temperature variation, namely ΔT_{Olsen}, and 2) the entropy

change associated to the removal of the electric field at the leg CD (electrocaloric effect). These can be expressed as

$$Q_1 = \rho c_P{}' \Delta T_{Olsen} \qquad (2)$$

$$Q_2 = \rho T_h \Delta S_{E\to 0} \qquad (3)$$

with $c_p{}'$ being the specific heat background value, measured experimentally with Differential Scanning Calorimetry (DSC) (SM note 3). Unfortunately, Q_2 cannot be measured quantitatively in our DSC because the mass of the PST-MLCs is too large (~300 mg vs the maximum of 20 mg that can be placed in the DSC). Hence, we assume $T_h \Delta S_{E\to 0} \cong c_P{}' \Delta T_{E\to 0}$, which is a very good approximation, as shown in [6]. Note that $\Delta T_{E\to 0}$ is the electrocaloric (EC) effect under adiabatic conditions. Thus, Q_{in} becomes

$$Q_{in} = \rho\, c_p{}'(\Delta T_{Olsen} + \Delta T_{EC}) \qquad (4)$$

with $c_p{}'$ = 300 J kg^{-1} K^{-1} and ρ = 8570 Kg m^{-3} (measured experimentally in our laboratory, SM note 3). To ensure an upper bond for Q_{in}, we take the highest value of ΔT_{EC} at 750 V applied. This is ΔT_{EC} = 3.10 ± 0.11 K (at T = 30 °C, SM Fig. 12.2). Under these conditions, the sample is in the ferroelectric phase before it is discharged (V = 750 V), and it transitions to the paraelectric phase once it is discharged (V = 0 V). The term $c_P' \Delta T_{E\to 0}$ is thus 986 J kg^{-1}, notably larger than the latent heat associated to the phase transition when crossed with temperature (L = 572 J kg^{-1}) because it includes the electric field contribution (dipolar entropy). We assume then $c_P' \Delta T_{E\to 0}$ = 986 J kg^{-1} constant to all points. This gives us an upper bond for Q_{in}, and thus a lower bond for efficiency. Note that the latent heat L = 572 J kg^{-1} does not vary with the electric field applied as our previous study on bulk PST has shown [6]. Instead, the effect of applying an electric field is to shift the transition temperature T_C towards higher temperatures.

It is also interesting to consider the upper bound Carnot efficiency that reads

$$\eta_{Carnot} = \frac{\Delta T}{T_{hot}} \quad (5)$$

Hence, from equations 1, 4 and 5, the relative efficiency η_r with respect to Carnot (scaled efficiency) is

$$\eta_r = \frac{N_d T_{hot}}{\rho c_p{}'(\Delta T_{Olsen} + \Delta T_{EC}) \Delta T_{Olsen}} \quad (6)$$

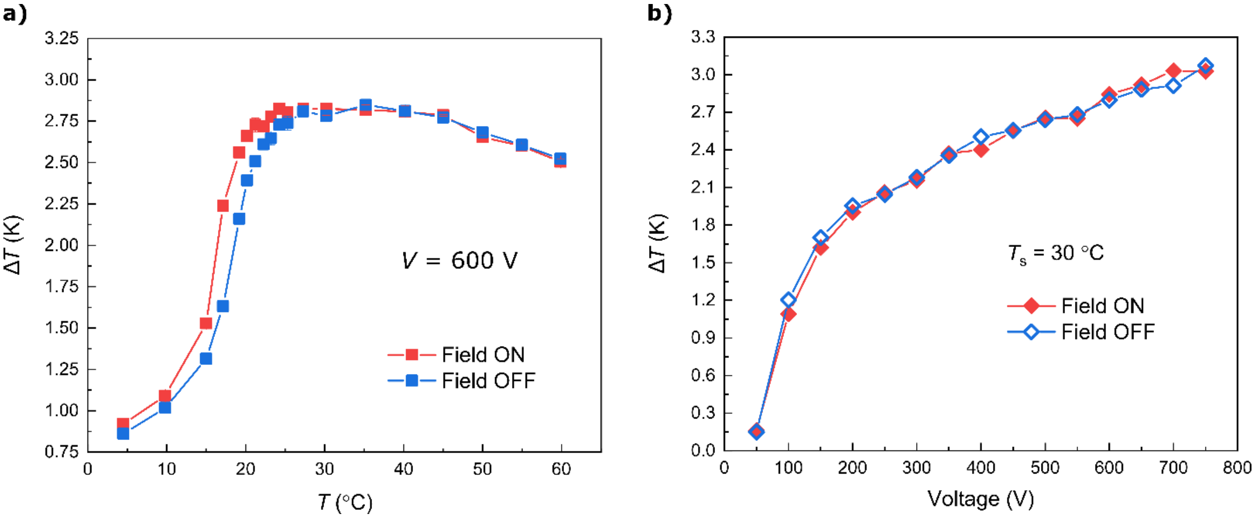

Supplementary Figure 12.2 Electrocaloric characterisation of 0.5 mm PST-MLCs as a function of temperature and 600 V (155 kV cm^{-1}) applied (**a**) and as a function of voltage and 30 °C (**b**).

Note 13. Capacitance versus temperature

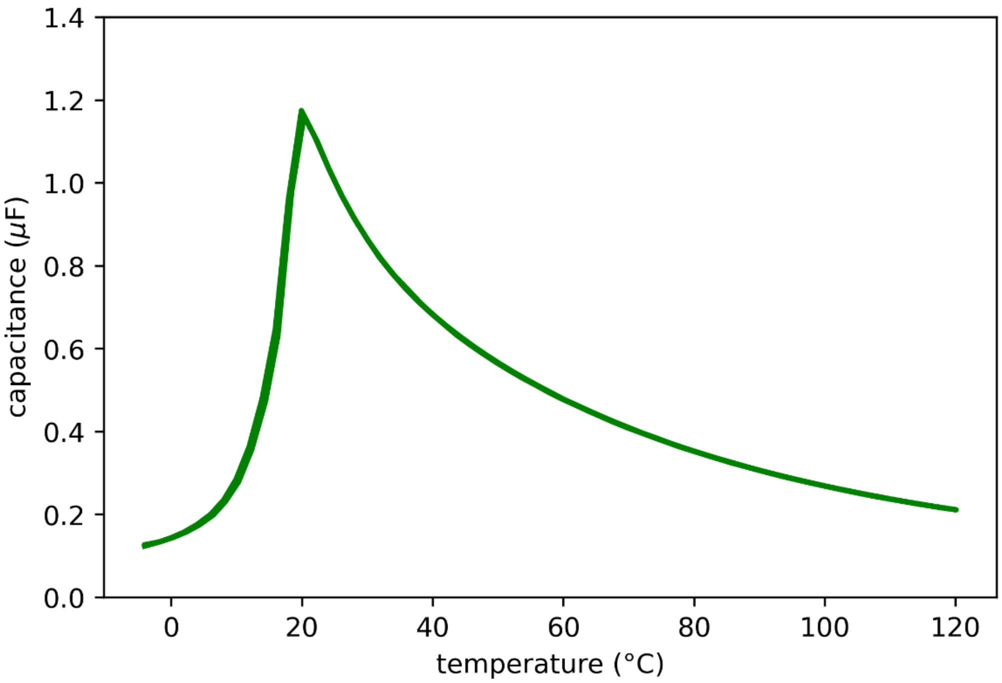

Supplementary Figure 13.1 – capacitance versus temperature of one 0.5 mm-thick MLC.

Measuring conditions: Aixacct tool measuring capacitance versus temperature at 500 Hz. The temperature has been varied from - 4°C to 120°C and then back to - 4°C.

The capacitance versus temperature clearly shows the phase transition that occurs in PST around 20°C (TC). This observation is perfectly in line with the measurements performed with Differential Scanning Calorimetry in Supplementary Note 3. Below TC, PST is ferroelectric. Beyond TC, PST is paraelectric. Below TC, there is a slight hysteresis between the data collected while heating and cooling, though the difference is barely visible.

Supplementary Table 1.

Maximum harvested energy density N_d in J cm^{-3} of representative examples from the literature performed on actual Olsen cycles at a given electric field *E,* initial temperature T_i and temperature span ΔT_{span}.

Material	Type - thickness (µm)	ΔT_{span} (K)	T_i (°C)	N_d (J cm^{-3})	*E* (kV cm^{-1})	Ref.
PZST	Ceramic - 250	20	145	0.12	32	[7]
0.85PMN-0.15PT	Ceramic – 1000	50	35	0.186	30	[8]
PZNT	Single crystal – 1000	60	100	0.24	20	[9]
60PVDF-40TrFE	Film - 50	75	25	0.52	400	[10]
PNZST	Ceramic – 500	70	150	0.78	13	[11]
8/65/35 PLZT	Ceramic - 290	135	25	0.9	75	[12]
0.68PMN–0.32PT	Thin film - 0.15	90	25	1.06	400	[13]
PST-MLC	MLC – 9 layers of 38 µm each	175	5	4.43	195	This work

PZST = $Pb_{0.99}Nb_{0.02}(Zr_{0.68},Sn_{0.25}Ti_{0.07})_{0.98}O_3$, PZNT = $Pb(Zn_{1/3}Nb_{2/3})_{0.955}Ti_{0.045}O_3$, PVDF = poly(vinylidene fluoride), TrFE = trifluoroethylene, PLZT = $(Pb,La)(Ti,Zr)O_3$, (1-x)PMN-yPT = $(1-x)Pb(Mg_{1/3}Nb_{2/3})O_3$ – x $PbTiO_3$, PNZST = $Pb_{0.99}Nb_{0.02}(Zr_{0.637}Sn_{0.273}Ti_{0.09})_{0.98}O_3$.

The one-shot energy density that we obtained on our pyroelectric modules in Olsen cycles (4.43 J cm^{-3}) can be directly compared with typical energy densities obtained with vibration energy harvesters. In Godard et al.[14], we compared piezoelectric materials in the context of vibration energy harvesters. The best one is based on lead zirconate titanate (PZT) and can harvest at maximum 80 mW cm^{-3} at 120 Hz. This stands for 6.5 10^{-4} J cm^{-3} per stroke, which means four orders of magnitude less than in our pyroelectric PST MLCs.

Supplementary Table 2

Parameters of the experimental macroscopic harvester HARV1.

ELEMENT	DIMENSIONS		
	Thickness (mm)	Length (mm)	Width (mm)
PST-MLC	1	7.2	10.4
Fluid slit	1	50.4	10.4
Harvester	9	50.4	10.4
# PST-MLC columns	7		
# PST-MLC rows	4		
# fluid slits	5		
Fluid flow (mL min^{-1})	112		
Voltage (V)	300-750		
Current (mA)	0-20		
Thermalization time (s)	26.5		
Waiting time (s)	2		
Period (s)	57		
Power (mW)	54.38		

Supplementary Table 3

Thermal properties of the materials used in the numerical modelling.

MATERIAL	c_P (J Kg^{-1} K^{-1})	k (W m^{-1} K^{-1})	ρ (kg m^{-3})
PST-MLC	300	2.1	8100
Silicone Oil	1634	0.16	913
Water	4180	0.6	1000

Supplementary Table 4

Parameters of the model that match the Harvester experimental performance.

ELEMENT	DIMENSIONS	
	Thickness (mm)	Length (mm)
PST-MLC	1	7.2
Fluid slit	1	50.4
Harvester	9	50.4
# PST-MLC columns	7	
# PST-MLC rows	4	
# fluid slits	5	
Fluid flow (mL min^{-1})	112	
Thermalization time (s)	25.2	
Waiting time (s)	2	
Period (s)	54.4	
Power (mW)	57	

Supplementary Table 5

Properties and dimensions of 1 mm thick PST-MLCs, taken from [2], and 0.5 mm ones, measured by us.

Property	0.5 mm thick PST-MLC	1 mm thick PST-MLC
Density (g cm^{-3})	8.6	8.8
Active area (mm^2)	48.7	49
Active layer thickness (mm)	0.386	0.379
# active layers	9	19
Active volume (mm^3)	16.9	35.3
Mass (g)	0.145	0.309
Specific heat (J kg^{-1} K^{-1})	300	290

References

[1] Y. Nouchokgwe, P. Lheritier, T. Usui, A. Torello, A. El Moul, V. Kovacova, T. Granzow, S. Hirose, E. Defay, *Scr. Mater.* **2022**, *219*, 114873.

[2] B. Nair, T. Usui, S. Crossley, S. Kurdi, G. G. Guzmán-Verri, X. Moya, S. Hirose, N. D. Mathur, *Nature* **2019**, *575*, 468.

[3] L. A. Shebanov, E. H. Birks, K. J. Borman, *Ferroelectrics* **1989**, *90*, 165.

[4] H.-C. Wang, W. A. Schulze, *J. Am. Ceram. Soc.* **1990**, *73*, 1228.

[5] Y. Podgorny, K. Vorotilov, A. Sigov, *AIP Adv.* **2016**, *6*, 95025.

[6] Y. Nouchokgwe, P. Lheritier, C.-H. Hong, A. Torelló, R. Faye, W. Jo, C. R. H. Bahl, E. Defay, *Nat. Commun.* **2021**, *12*, 1.

[7] R. B. Olsen, D. Evans, *J. Appl. Phys.* **1983**, *54*, 5941.

[8] G. Sebald, S. Pruvost, D. Guyomar, *Smart Mater. Struct.* **2007**, *17*, 15012.

[9] A. Khodayari, S. Pruvost, G. Sebald, D. Guyomar, S. Mohammadi, *IEEE Trans. Ultrason. Ferroelectr. Freq. Control* **2009**, *56*, 693.

[10] A. Navid, L. Pilon, *Smart Mater. Struct.* **2011**, *20*, 25012.

[11] J. Kim, S. Yamanaka, I. Murayama, T. Katou, T. Sakamoto, T. Kawasaki, T. Fukuda, T. Sekino, T. Nakayama, M. Takeda, M. Baba, H. Tanaka, K. Aizawa, H. Hashimoto, Y. Kim, *Sustain. Energy Fuels* **2020**, *4*, 1143.

[12] F. Y. Lee, S. Goljahi, I. M. McKinley, C. S. Lynch, L. Pilon, *Smart Mater. Struct.* **2012**, *21*, 25021.

[13] S. Pandya, J. Wilbur, J. Kim, R. Gao, A. Dasgupta, C. Dames, L. W. Martin, *Nat. Mater.* **2018**, DOI 10.1038/s41563-018-0059-8.

[14] N. Godard, L. Allirol, A. Latour, S. Glinsek, M. Gérard, J. Polesel, F. Domingues Dos Santos, E. Defay, *Cell Reports Phys. Sci.* **2020**, *1*, 100068.
